# Deciphering microRNAs and Their Associated Hairpin Precursors in a Non-Model Plant, *Abelmoschus esculentus*

**DOI:** 10.3390/ncrna3020019

**Published:** 2017-03-31

**Authors:** Kavitha Velayudha Vimala Kumar, Nagesh Srikakulam, Priyavathi Padmanabhan, Gopal Pandi

**Affiliations:** Department of Plant Biotechnology, School of Biotechnology, Madurai Kamaraj University, Madurai 625021, Tamil Nadu, India; kavithabt28@gmail.com (V.V.K K.); bioinagesh@gmail.com (S.N.); priyapadbhanabhan@gmail.com (P.P.)

**Keywords:** miRNAs, pre-miRNAs sequencing, *Abelmoschus esculentus*, next generation sequencing, non-model plant

## Abstract

MicroRNAs (miRNAs) are crucial regulatory RNAs, originated from hairpin precursors. For the past decade, researchers have been focusing extensively on miRNA profiles in various plants. However, there have been few studies on the global profiling of precursor miRNAs (pre-miRNAs), even in model plants. Here, for the first time in a non-model plant—*Abelmoschus esculentus* with negligible genome information—we are reporting the global profiling to characterize the miRNAs and their associated pre-miRNAs by applying a next generation sequencing approach. Preliminarily, we performed small RNA (sRNA) sequencing with five biological replicates of leaf samples to attain 207,285,863 reads; data analysis using miRPlant revealed 128 known and 845 novel miRNA candidates. With the objective of seizing their associated hairpin precursors, we accomplished pre-miRNA sequencing to attain 83,269,844 reads. The paired end reads are merged and adaptor trimmed, and the resulting 40–241 nt (nucleotide) sequences were picked out for analysis by using perl scripts from the miRGrep tool and an in-house built shell script for Minimum Fold Energy Index (MFEI) calculation. Applying the stringent criteria of the Dicer cleavage pattern and the perfect stem loop structure, precursors for 57 known miRNAs of 15 families and 18 novel miRNAs were revealed. Quantitative Real Time (qRT) PCR was performed to determine the expression of selected miRNAs.

## 1. Introduction

In plants, microRNAs (miRNAs) are 21–24 nucleotide (nt) in size and are crucial component of small RNAs (sRNAs). miRNAs are mostly transcribed by RNA Polymerase II to produce primary miRNAs (pri-miRNA). The stem-loop structure of pri-miRNA directs DCL1 (Dicer like 1) proteins to process near the base of the stem [1], resulting in the formation of precursor miRNA (pre-miRNA). The elimination of the pre-miRNA loop is subsequently followed by the continuous stretch of two- or multi-step cleavage at 21- nt intervals alongside the stem, resulting in the formation miRNA: miRNA* duplex. The precise miRNA recognition from the pri-miRNA relies on the precursor composition comprising processing signals such as 14–15-bp nt paired in stem proximal; a specific length of nt paired in stem distal to the miRNA: miRNA* duplex; and a terminal loop [2,3]. In addition, an alternative biogenesis pathway has been proposed: the loop-to-stem processing of pri-miRNAs [4]. The miRNA: miRNA* duplexes are methylated at their 3′ end by Hua Enhancer (HEN1) [5,6] to maintain their size and to defend from polyuridylation and consequent degradation [7]. The duplexes are channelized into cytoplasm, associated with Argonaute (AGO) protein to organize the RNA-induced silencing complex (RISC) where the miRNA* is sliced by the AGO at the 10th or 11th nt position [8]. The mature miRNA, in association with AGO, silences the target transcript either by direct cleavage (slicing) or by destabilization through slicer-independent turnover mechanisms and translational repression [9].

The emerging trend of next generation sequencing (NGS) technologies recently revealed various unknown genes and sRNAs in model and non-model plants [10,11]. sRNA sequencing in *Arabidopsis* exposed differentially expressed miRNAs along with other sRNAs during phosphate deficiency [12]. A report on the function of miRNAs in rice grain development by deep sequencing is available [13]. However, most of the studies are on mature miRNA profiling; efforts to explore the pre-miRNAs are limited, even in model plants. Unlike mature miRNA profiling, precursor miRNA profiling is not demanding due to its complicated hairpin structure and its low abundance [14]. Generally, Northern blot, in situ hybridization and Quantitative Real Time (qRT)-PCR had been used to resolve the pre-miRNAs expression pattern. To gain more insights into pre-miRNA sequences and secondary structure formation, rather than using sequencing approaches, researchers often prefer the genome sequences of corresponding miRNA: miRNA*. However, researchers may be left puzzled when miRNA* is absent.

Abelmoschus esculentus (lady’s finger or Abelmoschus), belongs to the *Malvaceae* family; allopolyploid in nature, it is cultivated around the globe in tropical, subtropical and warm temperate regions. *A. esculentus* is a vital vegetable crop, rich in fiber and vitamins with limited genome sequence information. sRNAs have proven to be regulators, targeting a large number of regulatory proteins. Apart from targeting various transcription factors, they also regulate protein stability, development, signaling pathways and plant pathogen resistance [15]. Hence, discovering the miRNAs’ role in *A. esculentus* will help us to understand the nexus between the miRNAs and their target genes, and throw light on the control of the *A. esculentus-*associated begomoviruses [16]. In this paper, to gain more insights into the miRNAs and their hairpin precursors, we executed NGS for miRNAs with five different *A. esculentus* leaf tissues. Data analysis applying miRPlant [17] identified 128 known miRNAs conserved across the plant kingdom and 845 novel miRNA candidates. To resolve pre-miRNAs for the known and novel predicted candidates, for the first time, we performed pre-miRNA sequencing by selecting libraries of size 160–300 (includes adaptors) nt. For precursor data analysis, we devised a strategy with stringent criteria and parameters by applying the Minimal Fold Energy Index (MFEI) and Dicer cleavage pattern with perfect stem loop structure. Eventually, we determined precursors for 18 novel miRNAs and 57 known miRNAs with high assurance.

## 2. Results

### 2.1. sRNA Sequencing Reveals Known as Well as Novel miRNA Candidates

**Figure 1:**
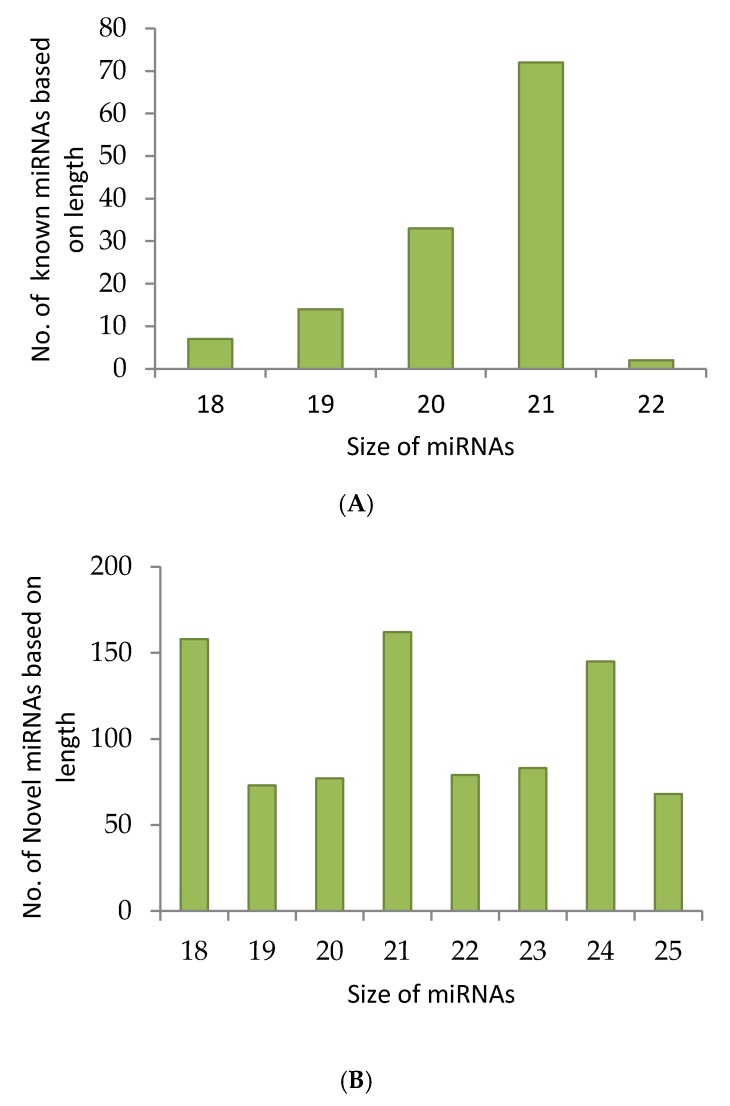

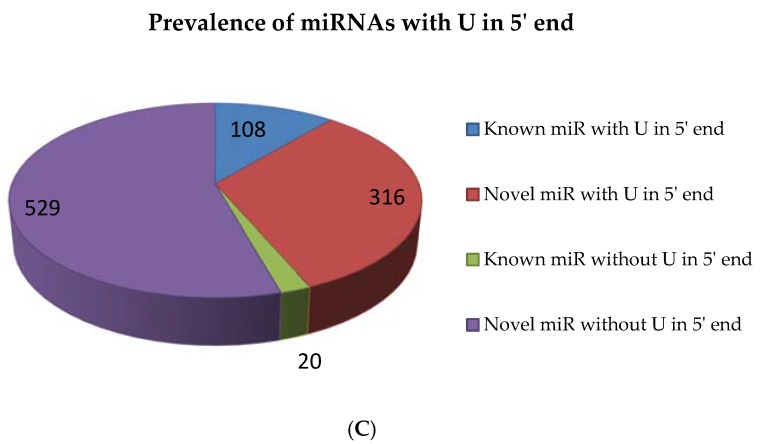
Small RNA (sRNA) sequencing data analysis in *Abelmoschus esculentus*. sRNA sequencing was performed and quality filtered data was analysed with miRPlant by using cotton, *Arabidopsis* and rice plants without allowing any mismatch. The filtered micro RNAs (miRNAs) were studied by investigating the terminal nucleotides (**C**), and the length of conserved (**A**) and novel (**B**) miRNAs.

For the identification of conserved and novel miRNAs in *A. esculentus*, five small RNA libraries were formed from the leaves by applying Illumina and Ion Torrent sequencing platforms. A total of 207,285,863 raw reads were gathered from all the five libraries. After adapter trimming, quality filtration was executed employing fastqc with a phred score of 30. Among the five datasets, two datasets were of poor quality and were consequently discarded. We proceeded with the analysis with the three datasets of approved quality. Quality filtration retained 99,229,831 reads, of which 8,188,118 were unique. The reads were subjected to transfer RNA (tRNA), ribosomal RNA (rRNA), small nuclear RNA (snRNA) and small nucleolar (snoRNA) removal and eventually 7,384,997 reads were retained for further analysis on miRplant [17] for miRNA prediction. The reads were mapped to the reference genome such as *Gossypium raimondii*, *Arabidopsis*, and *Oryza sativa* by not allowing any mismatch. miRPlant finds the flanking sequence around the reads and determines whether the genomic region forms a hairpin based on the RNA secondary structure algorithm. The scores for individual miRNAs are calculated by measuring the strength of the prediction. If the score for a predicted miRNA is higher, the probability of that miRNA being a true candidate is increasing [17]. Accordingly, miRNAs with positive scores were considered for miRNA candidates. miRPlant predicted 1023 novel miRNAs and 128 conserved miRNAs. The selection of unique sequences amidst the repeated sequences based on their lengths yielded 845 novel miRNAs. The 128 conserved miRNAs are grouped under 28 families. For most of the conserved miRNAs predicted by miRPlant, the mature miRNA sequence and its complimentary sequence (miRNA*) is also predicted to be found. All the predicted novel miRNAs were subjected to similarity search by submitting in miRBase (Version 21) [18] to ensure their novelty. The read count summary of sRNA data analysis is mentioned in Table S1. Overall, 21 nt miRNAs dominated in conserved as well as novel candidates followed by 20 nt miRNAs (Figure 1A,B). Among the 128 conserved miRNAs, 108 miRNAs carried U residue in their 5′ end and among the 845 novel miRNAs predicted, 316 miRNAs showed U residue in their 5′ end (Figure 1C).

### 2.2. Pre-miRNAs Profiling and Determination of Pre-miRNAs for Known and Novel miRNAs

As we identified 128 known and 845 novel miRNA candidates, we intend to reveal the sequence of pre-miRNAs for the identified miRNAs. Therefore, we designed a strategy to sequence the pre-miRNAs as mentioned in the methods section. The 160–300 nt libraries are size selected and subjected to Illumina sequencing in paired ends to attain 83,269,844 reads. Pre-miRNA mapping could not be performed due to a lack of *A. esculentus* genome sequence information.

Paired-end pre-miRNA sequencing reads were assembled as mentioned in the methods section, followed by quality filtration. Assembly yielded 41,194,311 reads. Quality filtration with a phred score cutoff value of 30 and adapters trimming retained 13,620,056 reads. A total of 386,003 reads were found to be unique. We carried out rRNA, tRNA, sn and snoRNA elimination and retained 147,028 reads. The 40–241 nt adapter trimmed sequences were considered as long reads for pre-miRNAs determinations.

**Figure 2:**
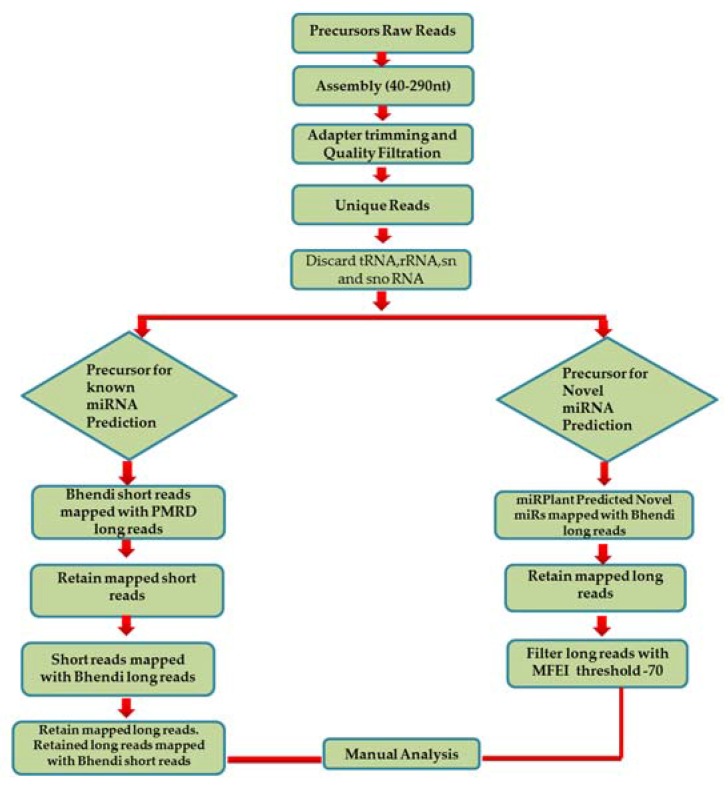

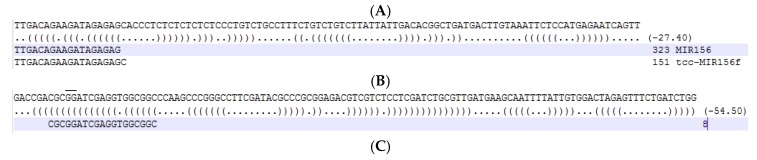
Pre-miRNA data analysis. In order to reveal the pre-miRNA sequences for the known and novel miRNAs, pre-miRNA sequencing was carried out and data was analysed in two ways: (**A**) By mapping the short reads (sRNA data) with the long reads (pre-miRNA data). The mapping patterns were studied by modifying the miRGrep protocol for known miRNAs (**B**) and novel miRNAs (**C**).

As we mapped the short reads on the long reads, we found a sliding pattern of short reads within a single long read at 1 nt interval which left us puzzled and meant that more effort would be required to analyse the data (Figure S1). Hence, we decided to analyse the data by retrieving known and novel miRNA reads from Plant micro RNA Database (PMRD) [19] and miRPlant by executing the below mentioned strategy (Figure 2A):

(I) In order to predict the precursors for conserved miRNAs, initially, 15,041 known pre-miRNA sequences retrieved from PMRD were considered as long reads. The short reads of *A*. *esculentus* were mapped with the long reads from PMRD to analyse the overlapping position by not allowing any mismatches; it was speculated that only conserved miRNA: miRNA* would be aligned with the PMRD long reads. A total of 5069 short reads showing alignment were extracted and mapped to *A*. *esculentus* pre-miRNA data to determine the pre-miRNA reads precisely. A total of 549 mapped long reads were retrieved and remapped with short reads for complete cluster formation. The predicted precursors were subjected to manual analysis to filter out the incomplete precursors and precursors whose mature miRNA falls in the loop region. Manual analysis retained only 25 complete precursors with perfect stem-loop structure, with the mature miRNA held in their stem region. The mapping summary for the three small RNA datasets is shown in the Table 1 and the mapping pattern of miRNA 156 with its precursor is shown in Figure 2B.

The number of reads obtained during the precursor data analysis was shown Table 1. The precursor reads were mapped with small RNA reads from datasets 1, 2 and 3. No- Number

(II) To predict the novel pre-miRNA candidates, the pre-miRNA reads mapped with the 845 novel miRNAs, predicted by miRPlant—were retrieved and aligned with the *A*. *esculentus* small RNA dataset to see the mature miRNA and its complimentary sequence clusters. The final results were subjected to manual analysis to reveal the mapping pattern of the novel miRNAs as depicted in Figure 2C.

We predicted the stem and loop structure for the selected long reads using RNAfold [20] and *p* value by Randfold [21]. We set the following criteria for determining the perfect precursors:

- The long reads which showed unambiguous secondary structure are considered as possible pre-miRNA candidates.
- The minimum length of the long reads chosen was 40 nt.
- We calculated the minimal fold energy (MFE) for the reads using RNA Fold and Randfold. The minimum fold energy index (MFEI) was calculated [22] as follows.

MFEI = (100 * MFE)/Length of RNA/(G + C)%

The possible pre-miRNA candidates had many splicing variants and incomplete precursors along with perfect stem loop structured precursors. To determine the perfect precursors, we carried out manual analysis using the following criteria.

- The base pairing between the mature miRNA and its complimentary sequence includes no more than four mismatches.
- The asymmetric bulges in between the duplex region are less frequent and should contain less than four bases. However, the total number of mismatches in the duplex region is no more than four.
- The precursors which carry the mature miRNA in its loop region are discarded. Also, the precursors with bigger loops in between the duplex region are also discarded.
- For novel precursors, we preceded with sequences holding the MFEI at −70 as their minimum threshold to discard other RNA species contamination such as rRNAs and tRNAs. Moreover, we manually checked the individual precursors by similarity searching in National Center for Biotechnology Information (NCBI) [23] to ensure that the sequences are devoid of these contaminating RNAs.
- In the case of novel precursors, some novel miRNAs are predicted based on their expression as well as their mapping pattern with the precursor.

**Figure 3:**
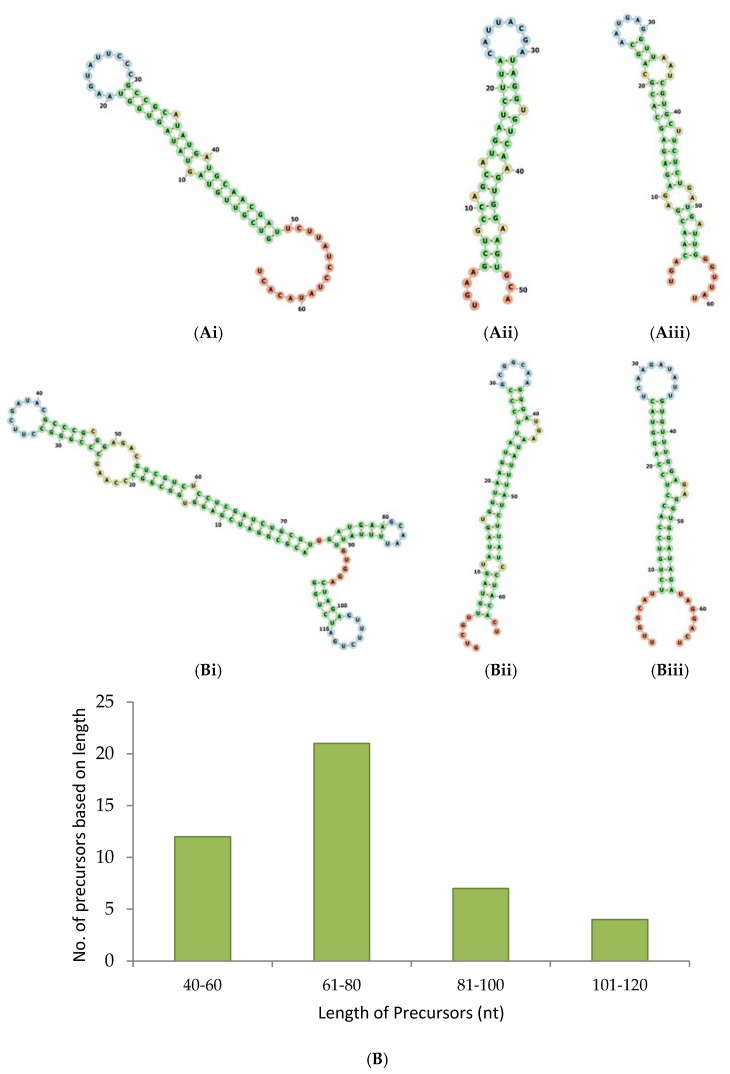

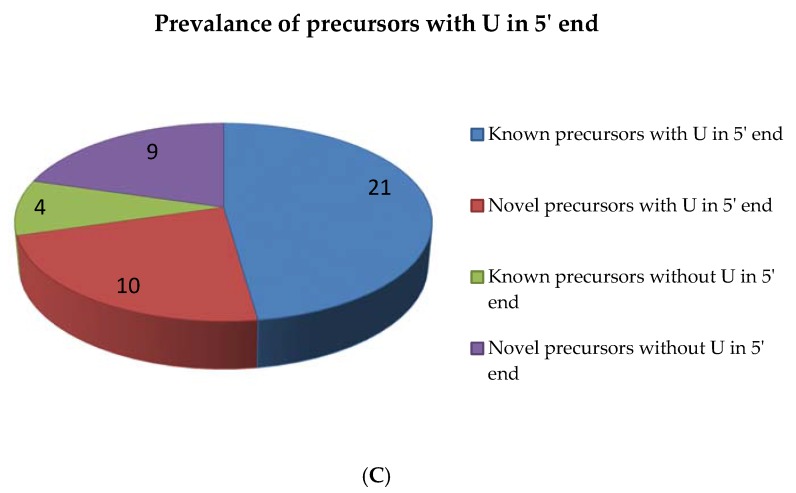
Secondary structure of pre-miRNAs. The selected pre-miRNAs were mapped to the rRNAs, tRNAs, sn and snoRNAs to ensure that they did not align with them. The pre-miRNAs’ MFE and MFEI were calculated and subjected to secondary fold formation for the known (**Ai**–**iii**) and novel (**Bi**–**iii**) miRNAs by using RNAFold [20]. In addition, pre-miRNA candidates were studied based on their length (**C**) and the presence of U terminal nuleotide (**D**).

Overall, we located precursors for 18 novel miRNAs and 57 conserved miRNAs of 15 families. The secondary structures of some of the known and novel miRNAs are depicted in Figure 3A,B.

### 2.3. Precursors of Conserved miRNAs and Novel miRNAs

Among the precursors for known and novel miRNAs, many precursors for a particular miRNA showed similarity in their 5′ end and slight variation in their 3′ end, indicating that they were splicing variants obtained during gene duplication events. Apart from these variants, we observed different precursors with the perfect stem loop structure with the mature miRNA and its complimentary sequence falling on the stem region. Precursors were abundant for the conserved miRNAs such as miRNA159, 6300 and 482 and also for the novel miRNA 3. Many of them were fragments of precursors and possible splicing variants. We chose only the perfect precursors by applying the above-mentioned criteria and found precursors for 15 conserved miRNA families, which include miRNA 157, 159, 482 and so on. We found many precursors for a single miRNA and vice versa. miRNA 482 and miRNA 159 have three and four precursors respectively. In addition, each of the miRNAs such as miRNA 6300, 396, 168 and 408 have two precursors separately, like novel miRNA 4. miRNA 157 and 156 share the same precursor, which is obvious as they share a high degree of sequence similarity. However, there is still a separate precursor for miRNA 156 alone which does not give rise to miRNA157. For most of the miRNAs, such as miRNA 166, 159, 156 and 160, a single precursor gave rise to many members in a particular family. Apart from these precursors, there are precursors particularly intended for miRNA159a, 482a, 159b and 166a, which are devoid of their other family members. Known miRNAs along with their precursors are listed in Table 2. We found precursors for seven novel miRNAs predicted by miRPlant. In addition, we predicted 11 novel miRNAs based on the mapping pattern with their respective precursors as well as their expression from the small RNA data. Surprisingly, we found that three of the novel miRNA precursors have their origin in the chloroplast DNA which demands further experimental validation. The novel miRNAs along with their precursors are listed in Table S2.

The MFEI of the precursor sequences ranges from −0.36 to −0.94 with an average of −0.72. The *p* value of the precursors ranges from 0.005–0.3 with an average of 0.04. Altogether, we found 44 precursors which include 25 precursors for known miRNAs and 19 precursors for novel miRNAs. The length of the precursor ranges from 42–114 nt (Figure 3C). Similar to mature miRNAs, we noted the prevalence of U residues in the 5′ end of the precursor sequences also. Among the 25 known miRNA precursors, 21 precursors showed U in their 5′ end. Among the 19 novel miRNA precursors, 10 precursors showed U in their 5′ end (Figure 3D)

### 2.4. Precursors of miRNAs from PMRD Not in miRBase

In addition to the conserved miRNAs submitted in miRBase, we found some miRNAs which are no longer submitted in miRBase but are available in PMRD, and aligned with our precursor data. We found precursors for seven such miRNAs, which include miRNA35-npr, miRNAf10238-npr, miRNAf10239-npr, miRNAf11010-npr, miRNAf11025-akr, miRNAf10082-akr and miRNAf10271-akr. These miRNAs mostly descended along with other miRNAs such as miRNA160, 168, 530, 396 and 159 from their respective precursors. For example, miRNAf 10238-npr arose along with the miRNA 160 precursor and miRNAf 10239-npr was produced along with the miRNA 168 precursor. Others include miRNAf 11025-akr and miRNAf 10082-akr with miRNA 530; miRNAf 11010-npr with miRNA 396; and miRNAf 10271-akr with miRNA 159. Apart from these precursors, there is a separate precursor for miRNA 35-npr which is particular to that miRNA alone. These miRNAs, along with their precursors, are listed in Table S3.

### 2.5. miRNAs from 5′ and 3′ Positions of Precursors

In any precursor, the mature miRNA descends either from the 5′ or 3′ position of the precursor. Most of our known miRNAs are produced from the 5′ position of their corresponding precursors, except the known miRNA 6424 whose position is in the 3′ end of the precursor. Among the novel miRNAs, 12 miRNAs have their position in the 5′ end and six miRNAs have their position in the 3′ end of their respective precursors. Though we had more than one precursor for some of the known and novel miRNAs, all the precursors for a particular miRNA produced the mature miRNA from a single position: either 5′ or 3′ and not both.

### 2.6. qRT-PCR

Initially, we examined the specificity of the primers for each miRNA by performing reverse transcription and PCR. Negative controls such as RNA control and minus template control ensured that the PCR product is not a primer dimer and also ensured that DNase treatment is appropriate. The PCR products were digested with specific restriction enzymes to confirm the presence of specific miRNAs. *Hinc*II digestion of miRNA 157 released 39bp and 7bp fragments. *BstN*I digestion of miRNA 166 produced fragments of size 33bp and 8bp. *Sac*I digestion of miRNA 159 produced fragments of size 30bp and 17bp. NmiRNA 19 is digested with the enzyme *Sau3A*I to confirm the PCR product (Figure S2).

**Figure 4:**
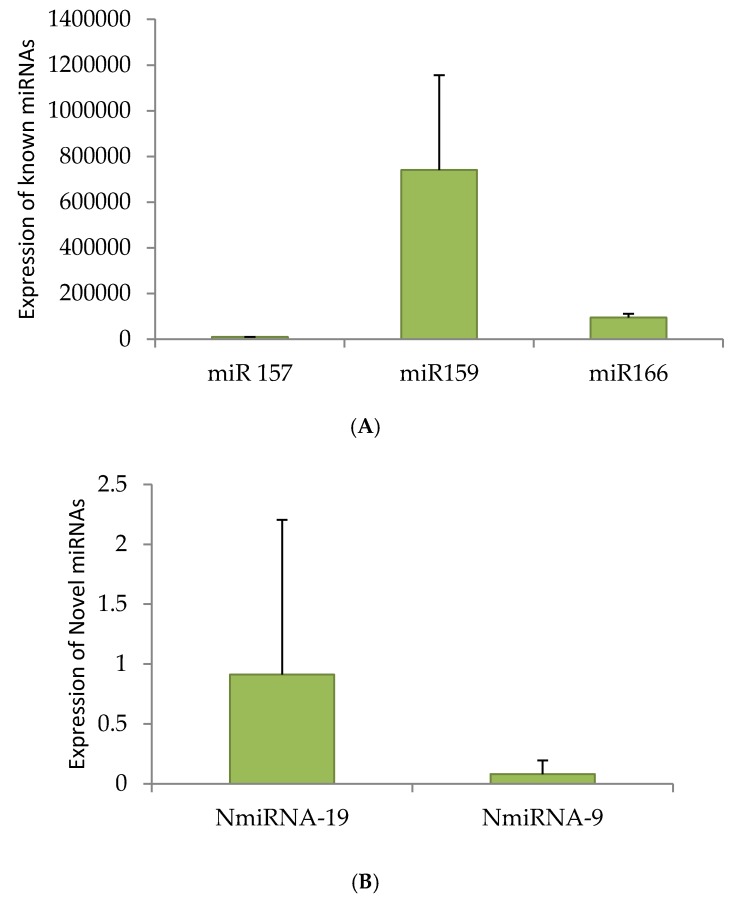
Quantitative Real Time (qRT)-PCR of miRNAs. Randomly selected miRNAs, miRNA-157, miRNA-159 and 166 (**A**) and two novel candidates (**B**) are reverse transcribed and confirmed the specific amplification by restriction digestion (Figure S2). Once genuine amplification is ensured, selected known and novel miRNAs were quantified by qRT-PCR.

qRT-PCR was performed for three known miRNAs—miRNA 166, 157 and 159—and for two novel miRNAs. miRNA 159 showed higher expression than the other two conserved miRNAs. NmiRNA 9 showed lower expression and on the other hand, the NmiRNA 19 showed higher expression (Figure 4A,B). The expression of miRNAs was normalised using 5.8S ribosomal RNA.

### 2.7. Target Prediction: 

Prediction of targets for novel miRNAs is necessary for its validation. Most of the targets predicted for miRNAs were transcription factors and genes involved in development, differentiation and metabolism. Targets are anticipated, both for conserved as well as novel miRNAs. Some of the targets predicted for known and novel miRNAs are mentioned in Table 3.

## 3. Discussion

In recent times, miRNAs have emerged as powerful regulators of gene expression at post-transcriptional or chromatin levels [24] which are exploited to reveal the fundamental mechanism of genes [25] and in the production of pathogen-resistant plants [26]. With the advent of next generation sequencing technology, researchers have become accustomed to finding the conserved as well as novel miRNAs in an astronomically immense number of plants, including non-model plants; some of them include *Arabidopsis* [10], tomato [27], cucumber [28], maize [29], rice [13], *Medicago* [30], *Citrus trifoliate* [31], *Hevea brasiliensis* [32], and potato [11]. miRPlant was used for the identification of conserved and novel miRNAs in cotton, which is taxonomically close to *Abelmoschus* [33]. In line with other studies, we performed sRNA sequencing and found 128 conserved known miRNAs and 845 novel miRNAs in *A. esculentus*. From the genome known plants, the maximum predicted miRNA number is 576 from soybean and rice*. G. raimondii* has only 294 miRNA candidates (miRBase, Version 21). Therefore, identifying 128 conserved miRNA candidates from the sRNA sequencing data is comparable and may be reliable.

Intriguingly, we predicted 845 novel candidates when we used cotton as a reference genome which is closely related to *A. esculentus*. The high numbers of novel miRNA predictions may depend on the chromosomal number as *A.esculentus* has a chromosomal number of 130 while for cotton it is 52. Though sRNA sequencing is common in plants, to the best of our knowledge, we did not find any precursor sequencing to date. Precursor miRNA sequencing has been performed in mice with locked nucleic acids to abstract other non-coding RNAs [34]. Moreover, precursor RNA sequencing was performed utilizing pre-miRNA-specific primers to enrich the pre-miRNAs [35]. In order to identify the precursor miRNAs for known and novel candidates in *A. esculentus* plants, we carried out pre-miRNA sequencing at a limit of 40–241 nt. The pre-miRNA length was restricted from 40–241 nt, keeping in mind the end goal to stay away from mRNA degradation products. Moreover, in plants, the stems of the precursor miRNAs are stable and conserved, and diversity is observed with the loop region and bulges [36]. Since the mature miRNA and miRNA* lie in the stem region, we aligned the reads gathered by sRNA sequencing to the precursor miRNA sequencing reads. Among the precursors of conserved miRNAs, we found several precursors sharing common sequences in the 5′ position but differing slightly in their 3′ position. These may be splicing variants of a particular miRNA gene. There are several miRNAs arising from one specific precursor. For example, miRNA 157 and 156 arise from the same precursor. On the other hand, we obtained more than one precursor for the miRNAs 482, 159 etc., as reported in rice [37]. In addition, we observed a single precursor that gave rise to two distinct miRNAs. Although we obtained numerous precursors for a single miRNA, we selected those precursor candidates that not only have the most effective mature miRNA sequence, but additionally must form the precise stem loop structure with the desired minimal fold energy index and *p* value. We discarded the reads which do not fulfil the criteria as mentioned within the methods section. For half of the known miRNAs from the sRNA data predicted in miRPlant, we obtained their corresponding precursors from the precursor data showing their relevance. Although we used novel miRNAs from miRPlant to obtain the precursors, we predicted some novel miRNAs from the precursor data as their mapping pattern shows that they fall in the stem region of the precursor with less than four mismatches.

Prediction of the secondary structure of pre-miRNAs and calculation of the free energy are required for the false positive reduction of precursor miRNAs [21]. The MFEI is an important criterion used in differentiating the precursors from the other RNAs, both coding and noncoding. In a precursor study in Asiatic cotton, it was found that the MFEI of the identified precursors fall in the range of 0.29–1.85 [38]. Most of our precursors have their MFEI value at around −0.72 since, in order to predict the novel precursors, we set a stringent criterion discarding all the other precursors which fall beyond the minimum threshold limit of −0.70. In a study in *Cassava*, the MFEI of the precursors was reported to be in the range of −0.84 to −1.20 [39]. It was reported that the MFEI for tRNA is 0.64, for mRNA is 0.65 and for rRNA is 0.59 [22]. Although a few of our precursor sequences fall within the above-mentioned MFEI, we confirmed whether they are contaminating RNAs by manually checking with NCBI-BLAST [23]. Thus, by following stringent criteria, we determined the pre-miRNAs sequence for 18 of the 845 novel miRNA candidates and further characterization of those candidates should be carried out. It is noteworthy that, out of 41 million assembled pre-miRNA reads, we observed less than 1% pre-miRNAs. The feasible logic explanation may be the rapid processing of pre-miRNA by Dicer and its co-proteins since pre-miRNAs serve as an intermediate during mature miRNA processing. In addition, the secondary hairpin structure of the precursor may affect the adapter ligation during library preparation, resulting in a limited number of reads.

## 4. Conclusions

*A. esculentus* is an important vegetable crop with little genome information. Using NGS technology, we have identified *A. esculentus-*associated miRNAs and their intermediate products which are known to play a key role in gene expression and plant resistance. This data will lay a foundation on which to unravel the miRNA: mRNA relationship which could be useful to develop pathogen-resistant plants.

## 5. Materials and Methods

### 5.1. RNA Extraction

RNA was extracted from the leaves of *A. esculentus* by slight modifications in the Trizol method to discard the mucilaginous content. A sample of 100 mg was minced in liquid nitrogen to a fine powder and 1.5 mL of Trizol, 250 μL of 100 mM Sodium citrate (pH-6) and 250 μL of 5 M sodium chloride were added. After brief mixing, the samples were allowed to stand for 5 min at room temperature followed by 200 μL of chloroform addition. The sample was shaken vigorously for 15 s and allowed to stand for 10 min at room temperature. The mixture was centrifuged at 14,000 rpm for 30 min at 4 °C and in the upper aqueous phase was transferred into a fresh tube. Precipitation was done with 100% isopropanol and the RNA quality was checked with Nanodrop ND-1000 (Thermo Scientific, Waltham, MA, USA) followed by electrophoresis. After DNase (Macherey Nagel, Düren,Germany) treatment, the quality was rechecked by the above-mentioned methods and the RNA was used for NGS or qRT-PCR.

### 5.2. sRNA Sequencing Libraries Preparation

Libraries of sRNAs were prepared by using True Seq Illumina sRNA library kits. Before library preparation, the RNA quality was checked by electrophoresis and Nano Drop ND-1000 (Thermo Scientific, Waltham, MA, USA) and the RNA integrity was assured by Bioanalyzer (Agilent, Santa Clara, CA, USA). We isolated leaves from five different plants to have biological replicates. The good quality RNA from five *A. esculentus* leaf samples was size fractionated ranging from 10–40 nt. Using RNA ligase (New England Biolabs, M0242, Ipswich, MA, USA), artificial adapters were ligated at the 3′ and 5′ end, reverse transcribed and amplified using Illumina sequencing primers or Ion Torrent primers. The first strand cDNA synthesis was carried out for 50 min at 65 °C using SuperScript III reverse transcriptase (Invitrogen, 18064014, Carlsbad, CA, USA). PCR was performed for fifteen cycles (98 °C for 10 s, 60 °C for 30 s and 72 °C for 15 s) and amplified products were subsequently cleaned and enriched by polyacrylamide gel electrophoresis (PAGE). Size selection of the library in the range of 140–160 bp was followed by overnight gel elution and salt precipitation utilizing Glycogen (Invitrogen, 10814-010), 3 M Sodium Acetate (Sigma, S7899, St. Louis, MO, USA) and absolute ethanol (Merck, 100983, Darmstadt, Germany) and the resulting precipitate was re-suspended in nuclease-free water. The libraries were quantified and quality was validated utilizing a Qubit Fluorometer and High Sensitivity Bioanalyzer Chip (Agilent, Santa Clara, CA, USA) respectively. For small RNA sequencing, a total of five samples were processed. Three samples were processed by Illumina sequencing platform (Genotypic Pvt. Ltd., Bangalore, India) and two samples were processed by Ion torrent sequencing platform (Shrimpex Biotech, Chennai, India).

### 5.3. sRNA Sequence Read Mapping

Small RNA raw reads were trimmed for their adapters followed by quality checking by FastQC [40]. Adapter trimming and quality filtration was executed using Trimmomatic [41]. FAST X Artifacts filter [42] has been used for filtering sequencing artefacts. We used fq trim [43] for phred score conversion. After quality filtration, other contaminating RNAs such as rRNA, tRNA, sn and snoRNAs are removed by alignment with bowtie [44]. The filtered small RNA sequences were analysed through miRPlant [17]. Since the *A. esculentus* genome sequence is not available, we used *G. raimondii*, *Arabidopsis* and *O. sativa* as reference genomes to find out the conserved as well as novel miRNAs by adopting the default parameters.

### 5.4. Precursor miRNA Library Preparation

Adopting the Illumina TruSeq Small RNA library protocol outlined in TruSeq Small RNA Sample Preparation Guide, the precursors RNA sequencing library was constructed. To 1 μg of total RNA 3′ adaptors were ligated followed by 5′ adaptor ligation. Reverse transcription of the ligated products was carried out by Superscript III Reverse transcriptase (Invitrogen, 18064014) after priming with reverse transcription primers. cDNA enrichment and barcoding by PCR (15 cycles, 98 °C for 10 s, 60 °C for 30 s and 72 °C for 15 s) were subsequently performed and products were cleaned by polyacrylamide gel. Libraries in the range of 160–300 bp (including adaptors) were size selected and gel eluted. Salt precipitation, quantity and quality checking were performed as mentioned in sRNA library preparation. Illumina sequencing (Illumina Next Seq 500, Genotypic Pvt. Ltd., Bangalore, India) was performed for two libraries in paired ends to cover the 160–300 nt sequences.

### 5.5. Data Analysis

Paired-end pre-miRNA sequencing reads were assembled by using Paired-End read merger (PEAR) [45]. Assembled pre-miRNA reads of length between 40–290 nt were retained. This contained sequences with or without adaptor sequences either in a single end or in both ends. After assembly, we performed adapter trimming and quality filtration with a phred score cutoff value of 30, followed by removal of rRNAs, tRNAs, sn and snoRNAs by mapping using bowtie2 [46]. The adapter trimmed 40–241 nt long reads are considered as possible pre-miRNA candidates, on which we analysed sRNA reads (18–34 nt) alignment by using soap.short without allowing any mismatch. From the miRGrep tool, we have used the unique_reads_for_mapping.pl, S1_reversed.pl and S0_print.pl scripts [14] to generate the mapping pattern files. We used RNA Fold and Randfold to calculate the minimum fold energy and infoseq [47] to calculate the Guanine Cytosine (GC) content. The possible pre-miRNA candidates were selected based on their length, MFEI, Dicer cleavage pattern and their perfect stem-loop structure. The criteria for pre-miRNA determination and parameters were discussed in detail in the results section.

### 5.6. qRT PCR

To quantify and validate sRNA sequencing, we did qRT-PCR [48]. Specific primers were designed for three known miRNAs such asmiRNA 166, 157 and 159—and two novel candidates to determine the expression. The primer list was mentioned in Table 4. For qRT-PCR, RNA was isolated by the modified Trizol method and DNase (Ambion, Austin, TX, USA) treated at 37 °C for 30 min. The RNA quality was checked in Nanodrop 1000 (Thermo Scientific, Waltham, MA, USA) and by electrophoresis. cDNA conversion was done as follows. Briefly, 100 ng of RNA was dissolved in a final volume of 10 μL including 0.1 mM of ATP, 1 μM of RT-primer, 0.1 mM of each deoxynucleotide (dATP, dCTP, dGTP and dTTP), 100 units of MuLV reverse transcriptase (New England Biolabs, Ipswich, MA, USA) and 1 unit of poly (A) polymerase (New England Biolabs, Ipswich, MA, USA). The sample was incubated at 42 °C for 1 h and enzyme inactivation at 95 °C for 5 min. Fast start Universal SYBR Green master (Roche, Basel, Switzerland) was used for quantification. qRT PCR was performed with four biological and experimental triplicates.

Before performing qRT-PCR, RT-PCR was performed with various controls to ensure that there was no primer dimer in the amplification. The amplified products of miRNA166, 167 and 159 were digested with *Sac*I, *Hinc*II and *BstN*I respectively and the novel miRNA (NmiRNA), NmiRNA 19, was digested with *Sau*3A I to confirm specific amplification. Once the genuine amplification was ensured, qRT-PCR was performed. Relative expression of miRNAs was quantified using the 2^−ΔΔc^T method [49].

### 5.7. Prediction of miRNA Target Genes

Target prediction was done using the psRNATarget tool [50]. *A*. *esculentus* transcriptome data [51] was used as the reference sequence. Targets were predicted using default tool parameters for known and novel miRNAs.

### 5.8. Accession Numbers

All the small RNA and precursor RNA raw data are submitted in NCBI under the BioProject accession ID-PRJNA352593.

## Figures and Tables

**Table 1:** Mapping summary of precursors with the small RNA datasets.

S. No	Precursors	Small RNA Dataset 1	Small RNA Dataset 2	Small RNA Dataset 3
1	No of precursors for known miRNAs	309	305	241
2	No of common precursors (for Known miRNAs) among the three datasets	219		
3	No of unique precursors (for known miRNAs) among the three datasets	330		
4	No of precursors for novel miRNAs	4863	4115	3509
5	No of common precursors (for novel miRNAs) among the three datasets	2003		
6	No of unique precursors (for novel miRNAs) among the three datasets	6793		

**Table 2:** List of known miRNAs and their precursors.

miRNA ID	miRNA Sequence	Precursor Sequence	MFEI
miR156	TTGACAGAAGATAGAGAG	TTGACAGAAGATAGAGAGCACCCTCTCTCTCTCTCCCTGTCTGCCTTTCTGTCTGTCTTATTATTGACACGGCTGATGACTTGTAAATTCTCCATGAGAATCAGTT	−0.696
miR482a	TCTTTCCTACTCCTCCCA	TCTTTCCTACTCCTCCCATTCCAGGGACGGAGGAGGCTAGGT	−0.750
miR6300	GTCGTTGTAGTATAGTGGT	GTCGTTGTAGTATAGTGGTAAGTATTCCCGCCGCATATGATGCAACGATTCTTATCCTATACACT	−0.659
miR157	TTGACAGAAGATAGAGAGCA	TTGACAGAAGATAGAGAGCACCCTCTCTCTCTCTCCCTGTCTGCCTTTCTGTCTGTCTTATTATTGACACGGCTGATGACTTGTAAATTCTCCATGAGAATCAGTT	−0.696
miR159	GGATTGAAGGGAGCTCTA	TTTGGATTGAAGGGAGCTCTATTCTGTGATGAAGCAATTTTATTGTGGACTAGAGTTTCTGATCTGG	−0.769
miR396	CACAGCTTTCTTGAACTT	TTCCACAGCTTTCTTGAACTTGCGAGTCAACGGGTTAGCAAACCCGCAAGGCGCAAGGAAGCTGATTGGCGGGATCCCTCGCGGGTGCACCGCCGA	−0.750
miR6424	TGGTGCCACGCTGTGTGCG	AGAGCTGAATGTGGTGTTTTGGGCTCATGCTTGCGTGGTGCCATCAAAACTTGGCATTGGAGAAGCGGTGATGGTGCCACGCTGTGTGCGACTGA	−0.696
miR168	TCGCTTGGTGCAGGTCGG	TCGCTTGGTGCAGGTCGGGAAATTACGATAGGTGTCAAGTGGAAGTGCA	−0.604
miR160	TGCCTGGCTCCCTGTATG	TGCCTGGCTCCCTGTATGCCACAATGTAGGCAAGGGAAGTCGGCAAAATGG	−0.775
miR530	TGCATTTGCACCTGCACC	TGCATTTGCACCTGCACCTTCTCATTACGATAGGTGTCAAGTGGAAGTGCA	−0.878
miR166	TCTCGGACCAGGCTTCAT	TCTCGGACCAGGCTTCATTCCCGAAGCCTGCCCAGCAGAACGACCCGCGAACGTGTTATCGAAAAAC	−0.463
miR535	CAACGAGAGAGAGCACGC	TGACAACGAGAGAGAGCACGCAGCAATGAGGTTAATCGTGCTTCTCTGATGATTGGGTTAT	−0.571
miR162	TCGATAAACCTCTGCATC	TCGATAAACCTCTGCATCCAGGAGCAATGAGGATAATCTGCTCTTGTGATGATAGGGTTATC	−0.881
miR408	CACTGCCTCTTCCCTGGCT	TGCACTGCCTCTTCCCTGGCTTTCAGGTCTCCAAGGTGAACAGCCTCTGGTCGATGGAACAATGTAGGCAAGGGAAGTCGGCAAAATG	−0.646
miR167	GCTGCCAGCATGATCTTA	TGAAGCTGCCAGCATGATCTTACATTACGATAGGTGTCAAGTGGAAGTGCA	−0.339

The known miRNA sequences along with their corresponding precursor sequences are shown. MFEI = minimum fold energy index.

**Table 3:** List of some known and novel miRNA targets predicted from *A*. *esculentus* transcriptome data.

S. No	miRNA	Target
1	miR-169	mitogen-activated protein kinase kinase kinase 1-like transcript
2	miR-166	Homeobox-leucine zipper family protein U-box domain-containing protein 26-like transcript calcium-transporting ATPase 10, plasma membrane-type-like, transcript variant casein kinase I-like transcript
3	miR-157	squamosa promoter-binding-like protein 2 LIGULELESS1 protein serine/threonine protein phosphatase 2A 55 kDa regulatory subunit B beta isoform-like transcript ubiquitin carboxyl-terminal hydrolase 9-like transcript Cyclin b1,5 isoform 1 transcript
4	miR-159	ABC transporter A family member 2-like transcription factor GAMYB-like U-box domain-containing protein 44-like forkhead box protein P2-like CBL-interacting serine/threonine-protein kinase 14-like
5	NmiRNA-4	ABC transporter A family member 2-like transcript transcription factor GAMYB-like transcript
6	NmiRNA-1	E3 ubiquitin-protein ligase RHA2A, mRNA
7	NmiRNA-7	copper-transporting ATPase RAN1, mRNA
8	NmiRNA-18	GBF-interacting protein 1-like, mRNA

**Table 4:** List of primers used for quantitative Real Time PCR.

S. No	miRNA	Forward Primer	Reverse Primer
1	miRNA159a	GCAGTTTGGATTGAAGGGA	AGTCCAGTTTTTTTTTTTTTTTAGAGC
2	miRNA 166a	GTCGGACCAGGCTTCAT	CCAGTTTTTTTTTTTTTTTGGGGA
3	miRNA 157a	CGCAGTTGACAGAAGATAGAG	TCCAGTTTTTTTTTTTTTTTGTGCT
4	NmiRNA 19	GGCGCAGAGTTACTAATTCATGA	GTCCAGTTTTTTTTTTTTTTTCAGAT
5	NmiRNA 9	CGCAGGGTGGCTGTAGTTTA	GTCCAGTTTTTTTTTTTTTTTACCAC

The forward and reverse primers for the corresponding known and novel miRNAs are shown.

## Supplementary Materials

Supplementary data are available online at www.mdpi.com/2311-553X/3/2/19/s1, Figure S1: Sliding pattern of Small RNA Reads within a precursor. Figure S2: Reverse Transcription PCR and restriction digestion of miRNAs. Table S1: Summary of small RNA data analysis. Table S2: List of Novel miRNAs and their precursors. Table S3: List of Precursors of miRNAs from PMRD which are not present in miRbase.

## References

[1] Bartel D.P. (2004). MicroRNAs: Genomics, Biogenesis, Mechanism, and Function. Cell.

[2] Mateos J.L., Bologna N.G., Chorostecki U., Palatnik J.F. (2010). Identification of MicroRNA Processing Determinants by Random Mutagenesis of Arabidopsis MIR172a Precursor. Curr. Biol..

[3] Werner S., Wollmann H., Schneeberger K., Weigel D. (2010). Structure Determinants for Accurate Processing of miR172a in Arabidopsis thaliana. Curr. Biol..

[4] Czech B., Hannon G.J. (2011). Small RNA sorting: Matchmaking for Argonautes. Nat. Rev. Genet..

[5] Li J., Yang Z., Yu B., Liu J., Chen X. (2005). Methylation Protects miRNAs and siRNAs from a 3′-End Uridylation Activity in Arabidopsis. Curr. Biol..

[6] Yu B., Yang Z., Li J., Minakhina S., Yang M., Padgett R.W., Steward R., Chen X. (2005). Methylation as a crucial step in plant microRNA biogenesis. Science.

[7] Zhao Y., Mo B., Chen X. (2012). Mechanisms that impact microRNA stability in plants. RNA Biol..

[8] Mallory A., Vaucheret H. (2010). Form, Function, and Regulation of ARGONAUTE Proteins. Plant Cell..

[9] Voinnet O. (2009). Origin, Biogenesis, and Activity of Plant MicroRNAs. Cell.

[10] Fahlgren N., Howell M.D., Kasschau K.D., Chapman E.J., Sullivan C.M., Cumbie J.S., Givan S.A., Law T.F., Grant S.R., Dangl J.L. (2007). High-Throughput Sequencing of Arabidopsis microRNAs: Evidence for Frequent Birth and Death of MIRNA Genes. PLoS ONE.

[11] Zhang R., Marshall D., Bryan G.J., Hornyik C. (2013). Identification and Characterization of miRNATranscriptome in Potato by High-Throughput Sequencing. PLoS ONE.

[12] Hsieh L.C., Lin S.I., Shih A.C.C., Chen J.W., Lin W.Y., Tseng C.Y., Li W.H., Chiou T.J. (2009). Uncovering Small RNA-Mediated Responses to Phosphate Deficiency in Arabidopsis by Deep Sequencing. Plant Physiol..

[13] Zhu Q.H., Spriggs A., Matthew L., Fan L., Kennedy G., Gubler F., Helliwell C. (2008). A diverse set of microRNAs and microRNA-like small RNAs in developing rice grains. Genome Res..

[14] Li N., You X., Chen T., Mackowiak S.D., Friedlander M.R., Weigt M., Du H., Gogol-Doring A., Chang Z., Dieterich C. (2013). Global profiling of miRNAs and the hairpin precursors: Insights into miRNA processing and novel miRNA discovery. Nucleic Acids Res..

[15] Rhoades M.W.J., Bartel D.P., Bartel B. (2006). Micro RNAs and Their Regulatory roles in Plants. Annu. Rev. Plant Biol..

[16] Priyavathi P., Kavitha V., Gopal P. (2016). Complex nature of infection associated with yellow vein mosaic disease in Bhendi (*Abelmoschus esculentus*). Curr. Sci..

[17] An J., Lai J., Sajjanhar A., Lehman M.L., Nelson C.C. (2014). miRPlant: An integrated tool for identification of plant miRNA from RNA sequencing data. BMC Bioinform..

[18] Kozomara A., Griffiths-Jones S. (2014). miRBase: annotating high confidence microRNAs using deep sequencing data. Nucleic Acids Res..

[19] Zhang Z., Yu J., Li D., Zhang Z., Liu F., Zhou X., Wang T., Ling Y., Su Z. (2010). PMRD: plant microRNA database. Nucleic Acids Res..

[20] Hofacker I.L. (2003). Vienna RNA secondary structure server. Nucleic Acids Res..

[21] Bonnet E., Wuyts J., Rouzé P., Peer Y.V. (2004). Evidence that microRNA precursors, unlike other non-coding RNAs, have lower folding free energies than random sequences. Bioinformatics.

[22] Zhang B.H., Pan X.P., Cox S.B., Cobb G.P., Anderson T.A. (2006). Evidence that miRNAs are different from other RNAs. Cell. Mol. Life. Sci..

[23] Altschul S.F., Gish W., Miller W., Myers E.W., Lipman D.J. (1990). Basic local alignment search tool. J. Mol. Biol..

[24] Moazed D. (2009). Small RNAs in transcriptional gene silencing and genome defence. Nature.

[25] Aukerman M.J., Sakai H. (2003). Regulation of Flowering Time and Floral Organ Identity by a MicroRNA and Its APETALA2 like Target Genes. Plant Cell.

[26] Qu J., Ye J., Fang R. (2007). Artificial Micro-RNA mediated Virus Resistance in Plants. J. Virol..

[27] Moxon S., Jing R., Szittya G., Schwach F., Pilcher R.L.R., Moulton V., Dalmay T. (2008). Deep sequencing of tomato short RNAs identifies microRNAs targeting genes involved in fruit ripening. Genome Res..

[28] Martinez G., Forment J., Llave C., Pallas V., Gomez G. (2011). High-Throughput Sequencing, Characterization and Detection of New and Conserved Cucumber miRNAs. PLoS ONE.

[29] Wang L., Liu H., Li D., Chen H. (2011). Identification and characterization of maize microRNAs involved in the very early stage of seed germination. BMC Genom..

[30] Briere C.L., Naya L., Sallet E., Calenge F., Frugier F., Hartmann C., Gouzy J., Crespi M. (2009). Genome-Wide Medicago truncatula Small RNA Analysis Revealed Novel MicroRNAs and Isoforms Differentially Regulated in Roots and Nodules. Plant Cell.

[31] Song C., Wang C., Zhang C., Korir N.K., Yu H., Ma Z., Fang J. (2010). Deep sequencing discovery of novel and conserved microRNAs in trifoliate orange (Citrus trifoliata). BMC Genom..

[32] Gebelin V., Argout X., Engchuan W., Pitollat B., Duan C., Montoro P., Leclercq J. (2012). Identification of novel microRNAs in Hevea brasiliensis and computational prediction of their targets. BMC Plant Biol..

[33] Naoumkina M., Thyssen G.N., Fang D.D., Hinchliffe D.J., Florane C.B., Jenkins J.N. (2016). Small RNA sequencing and degradome analysis of developing fibers of short fiber mutants Ligon-lintles-1 (Li1) and −2 (Li2) revealed a role for miRNAs and their targets in cotton fiber elongation. BMC Genom..

[34] Burroughs A.M., Kawano M., Ando Y., Daub C.O., Hayashizaki Y. (2011). pre-miRNA profiles obtained through application of locked nucleic acids and deep sequencing reveals complex 5′/3′ arm variation including concomitant cleavage and polyuridylation patterns. Nucleic Acids Res..

[35] Newman M.A., Mani V., Hammond S.M. (2011). Deep sequencing of microRNA precursors reveals extensive 3′ end modification. RNA.

[36] Xuan P., Guo M.Z., Wang J., Wang C.Y., Liu X.Y., Liu Y. (2011). Genetic algorithm-based efficient feature selection for classification of pre-miRNAs. Genet. Mol. Res..

[37] Lacombe S., Nagasaki H., Santi C., Duval D., Piegu B., Bangratz M., Breitler J., Guiderdoni E., Brugidou C., Hirsch J. (2008). Identification of precursor transcripts for 6 novel miRNAs expands the diversity on the genomic organisation and expression of miRNA genes in rice. BMC Plant Biol..

[38] Wang M., Wang Q., Wang B. (2012). Identification and Characterization of MicroRNAs in Asiatic Cotton (*Gossypium arboreum* L.). PLoS ONE.

[39] Rogans S.J., Rey C. (2016). Unveiling the Micronome of Cassava (Manihot esculenta Crantz). PLoS ONE.

[40] Andrews S. FastQC: A Quality Control Tool for High Throughput Sequence Data. http://www.bioinformatics.babraham.ac.uk?/projects/fastqc/.2010.

[41] Bolger A.M., Lohse M., Usadel B. (2014). Trimmomatic: A flexible trimmer for Illumina Sequence Data. Bioinformatics.

[42] Fast X Artefacts Filter. http://hannonlab.cshl.edu/fastx_toolkit/index.html.

[43] Fqtrim. http://ccb.jhu.edu/software/fqtrim/.

[44] Langmead B., Trapnell C., Pop M., Salzberg S.L. (2009). Ultrafast and memory-efficient alignment of short DNA sequences to the human genome. Genome Biol..

[45] Zhang J., Kobert K., Flouri T., Stamatakis A. (2013). PEAR: A fast and accurate Illumina Paired-End reAd merger. Bioinformatics.

[46] Langmead B., Salzberg S.L. (2013). Fast gapped-read alignment with Bowtie 2. Nat. Methods.

[47] Rice P., Longden I., Bleasby A. (2000). EMBOSS: The European Molecular Biology Open Software Suite. Trends Genet..

[48] Balcells I., Cirera S., Busk P.K. (2011). Specific and sensitive quantitative RT-PCR of miRNAs with DNA primers. BMC Biotechnol..

[49] Livak K.J., Schmittgen T.D. (2001). Analysis of Relative Gene Expression Data Using RealTime Quantitative PCR and the 2^−ΔΔc^T Method. Methods.

[50] Dai X., Zhao P.X. (2011). psRNATarget: A plant small RNA target analysis server. Nucleic Acids Res..

[51] Schafleitner R., Kumar S., Lin C., Hegde S.G., Ebert A. (2013). The okra (*Abelmoschus esculentus*) transcriptome as a source for gene sequence information and molecular markers for diversity analysis. Gene.

