# Supplementary material for: Deciphering microRNAs and Their Associated Hairpin Precursors in a Non-Model Plant, Abelmoschus esculentus
Source: Noncoding RNA. 2017 Mar 31;3(2):19. doi: 10.3390/ncrna3020019 (PMC5831935; doi:10.3390/ncrna3020019)
Supplement: Supplementary file 1 [file ncrna-03-00019-s001.pdf]

non-coding RNA

MDPI

Article

Deciphering microRNAs and Their Associated Hairpin Precursors in a Non-Model Plant, *Abelmoschus esculentus*

Kavitha Velayudha Vimala Kumar †, Nagesh Srikakulam †, Priyavathi Padmanabhan and Gopal Pandi *

Supplementary Fig. S1. Sliding pattern of Small RNA Reads within a precursor.

```
seq144534__1
TAGACCGACGCGGATCGAGGTGGCGGCCCAAGCCCGGGCCTTCGATACGCCCGCGGAGACGTCGTCTCCTCGATCGT
............(((((((.((((((.....(((((((.........))))).))....)))))).))))))).. (-35.90)
  GACCGACGCGGATCGAGG                                                          5
  GACCGACGCGGATCGAGGT                                                         5
  GACCGACGCGGATCGAGGTG                                                        1
  GACCGACGCGGATCGAGGTGG                                                       3
  GACCGACGCGGATCGAGGTGGC                                                      5
  GACCGACGCGGATCGAGGTGGCG                                                     22
  GACCGACGCGGATCGAGGTGGCGG                                                    8
  GACCGACGCGGATCGAGGTGGCGGC                                                   3
  GACCGACGCGGATCGAGGTGGCGGCC                                                  14
  GACCGACGCGGATCGAGGTGGCGGCCC                                                 2
  GACCGACGCGGATCGAGGTGGCGGCCCAAGC                                             2
  GACCGACGCGGATCGAGGTGGCGGCCCAAGCC                                            6
  GACCGACGCGGATCGAGGTGGCGGCCCAAGCCC                                           2
   ACCGACGCGGATCGAGGTG                                                        7
   ACCGACGCGGATCGAGGTGG                                                       4
   ACCGACGCGGATCGAGGTGGC                                                      13
   ACCGACGCGGATCGAGGTGGCG                                                     22
   ACCGACGCGGATCGAGGTGGCGG                                                    12
   ACCGACGCGGATCGAGGTGGCGGC                                                   1
   ACCGACGCGGATCGAGGTGGCGGCC                                                  3
   ACCGACGCGGATCGAGGTGGCGGCCC                                                 6
   ACCGACGCGGATCGAGGTGGCGGCCCAA                                               2
   ACCGACGCGGATCGAGGTGGCGGCCCAAGC                                             2
   ACCGACGCGGATCGAGGTGGCGGCCCAAGCC                                            1
   ACCGACGCGGATCGAGGTGGCGGCCCAAGCCC                                           5
   ACCGACGCGGATCGAGGTGGCGGCCCAAGCCCG                                          2
    CCGACGCGGATCGAGGTG                                                        42
    CCGACGCGGATCGAGGTGG                                                       19
    CCGACGCGGATCGAGGTGGC                                                      18
    CCGACGCGGATCGAGGTGGCG                                                     105
    CCGACGCGGATCGAGGTGGCGG                                                    32
    CCGACGCGGATCGAGGTGGCGGC                                                  54
    CCGACGCGGATCGAGGTGGCGGCC                                                 1634
    CCGACGCGGATCGAGGTGGCGGCCC                                                514
    CCGACGCGGATCGAGGTGGCGGCCCA                                               1
    CCGACGCGGATCGAGGTGGCGGCCCAAG                                             2
    CCGACGCGGATCGAGGTGGCGGCCCAAGC                                            6
    CCGACGCGGATCGAGGTGGCGGCCCAAGCC                                           11
    CCGACGCGGATCGAGGTGGCGGCCCAAGCCC                                          15
    CCGACGCGGATCGAGGTGGCGGCCCAAGCCCG                                         1
    CCGACGCGGATCGAGGTGGCGGCCCAAGCCCGG                                        1
     CGACGCGGATCGAGGTGGCGGC                                                  45
     CGACGCGGATCGAGGTGGCGGCC                                                 122
     CGACGCGGATCGAGGTGGCGGCCC                                                86
     CGACGCGGATCGAGGTGGCGGCCCA                                               4
     CGACGCGGATCGAGGTGGCGGCCCAA                                              6
```

Fig. S1. Sliding pattern of Small RNA Reads within a precursor. Short reads from small RNA sequencing were mapped to the long reads of precursor sequencing by using perl scripts from miRGrep along with in-house built shell scripts. A sliding pattern of small reads was observed for many precursors as illustrated in figure S1.

non-coding RNA

MDPI

Supplementary Fig. S2. Reverse Transcription PCR and restriction digestion of miRNAs

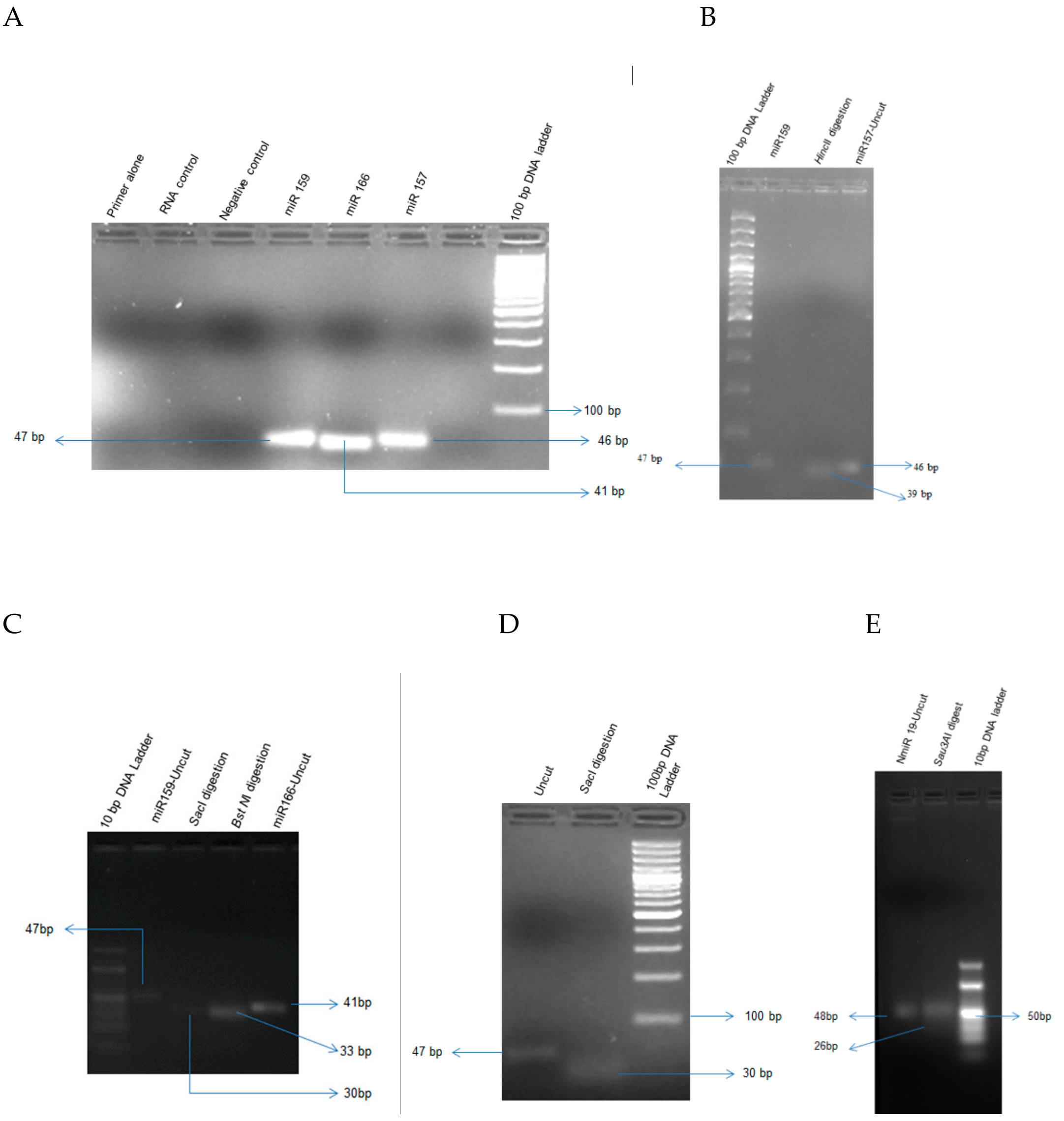

Fig. S2. Reverse transcription PCR and restriction digestion of miRNAs. PCR amplification was carried out for the randomly selected miRNAs from the reverse transcribed RNA (A). Amplified products were eluted and digested with specific restriction enzymes: *Hinc*II digestion for miRNA157 (B); *BstN*I digestion for miRNA166 (C); *Sac*I digestion for miRNA159 (D); and *Sau3A*I

digestion for NmiRNA19 (E), to ensure genuine amplification prior to qRT-PCR. All gels are 2.5%.

Table S1. List of Novel miRNAs and their precursors

miRNA ID	miRNA Sequence	Precursor Sequence	MFEI
NmiRNA1	TCTTTCCTACTCCTCCCAT	TCTTTCCTACTCCTCCCATTCCAGGGACGGAGGAGGCTAGGT	-0.750038
NmiRNA2	GCAAGGCGCAAGGAAGCTG	TTCCACAGCTTTCTTGAACTTGCGAGTCAACGGGTTAGCAAACCCGCAAGGCGCAAGGAAGCTGATTGGCGGGATCCCTCGCGGGTGCACCGCCGA	-0.750814
NmiRNA3	CGCGGATCGAGGTGGCGGC	ACGCGGATCGAGGTGGCGGCCCAAGCCCGGGCCTTCGATACGCCCGCGGAGACGTCGTCTCCTCGATCTGCGTTGATGAAGCAATTTTATTGTGGACTAGAGTTTCTGATCTGG	-0.756122
NmiRNA4	TTTGGATTGAAGGGAGCTCTA	TTTGGATTGAAGGGAGCTCTATGCCATGGTGGTCCTGCAGAGATTATCGAACCTGGTATATCAGGGTTCCATATTGATCCA	-0.767551
NmiRNA5	TATTTCCCCAGATGATCGA	TCCATTGTCGTCCAGTGGTTAGGATATCTGGGGAAGTGATGATTATTTCCCCAGATGATCGAAGCTGAACAAACTGTGCGATTTT	-0.905393
NmiRNA6	TGTAGTATAGTGGTAAGTATTC	GTCGTTGTAGTATAGTGGTAAGTATTCCCGCGGCAAGGGATGAATATTTATCTTATCCTACACT	-0.715473
NmiRNA7	TTGTAGTATAGTGGTGAGTAT	GTCGTTGTAGTATAGTGGTGAGTATTCCTGGCAAGAGATGAATACTTATTCTATCCTACACT	-0.770827
NmiRNA8	TGTGTTTGGAGAGGTGGAT	TTGGCATTCTGTCCACCTCCAGGTACTCAAGATATTGTGTTTGGAGAGGTGGATAGATAGGACT	-0.813838
NmiRNA9	GTGGCTGTAGTTTAGTGG	GGTGGCTGTAGTTTAGTGGTGAGAATTCCACGTTGTTGGAATTCTCGGGTGCCAAGGAACTCCAGTCACGTCCGC	-0.827552
NmiRNA10	ATGTAGCCAAGTGGATCAAGGC	TGTTTTCTCTGCCTTGATCTATATGCGCATGGCGGATGTAGCCAAGTGGATCAAGGCAGTGG	-0.867742
NmiRNA11	TCGATAAACCTCTGCATCCAG	TCGATAAACCTCTGCATCCAGGAGCAATGAGGATAATCTGCTCTTGTGATGATAGGGTTATC	-0.881449
NmiRNA12	CTTTTCTTTTTCTTATCC	CTTTTCTTTTTCTTATCCTACATTTGAGGATGATGAAAAAGATCAGACGGAT	-0.941243
NmiRNA13	GACCAAAGAGTCGGAGGGGG	ACCAGCATTCTTAAGACCAAAGAGTCGGAGGGGGGAAAGCTCTCCGTTCCTGGTTCTCCTGTAGCTGGA	-0.700042

NmiRNA14	TTCGTC CCTCTA ATATGT	TCTTTTATGGTTCATATTCTGGATTGGGTTCGTCCCTCTAATATGTAATATAAG ATGTAATAATGGGATGAACTAAGTTATAGGCATGAAAGTGTAAGAACTCAAC GG	-0.702688
NmiRNA15	ACTGG AGGTCT GTAGTT CG	TAAACTGGAGGTCTGTAGTTCGATCCTGCATGGGGGCACCTGGAATTCTCAGG TGCCAAGGAACTCCAGTCACGTCCGC	-0.704497
NmiRNA16	TTCGAT ACGCC CGCGG AGA	ACGTTCCCGTGGGCGGATCGAGGTGGCGGCCCAAGCCCGGGCCTTCGATACG CCCGCGGAGACGTCGTCTCCTCGATCGT	-0.744643
NmiRNA17	AGACG AAGCG GAAGC GAG	CCCTTTGTCGCTTCGATTCGTCGTTCCGCCTTAGGGGGAAGCACCCGCGTGAG CGGGAGTAGACGAAGCGGAAGCGAGAATGT	-0.746012
NmiRNA18	CCAGA TATAG AGATG GCGAC	AAACCAGATATAGAGATGGCGACTAAGGTTGCTGTTTCCATTATTATATAATT TCAAGATCACAATGGATCTATGA	-0.748108

Novel miRNA sequences along with their corresponding precursor sequences are shown. NmiRNA denotes- Novel miRNA.

Table S2. List of Precursors of miRNAs from PMRD which are not present in miRBase

miRNA ID	miRNA Sequence	Precursor Sequence
MIR35-npr	GGAATATGAGTG TGTGACTT	GGTAGTTCGACCGCGAAATTTATTTGTTTCTGTATTTCCGGAATA TGAGTGTGTGACTTGTTAGAATTGATC
MIRf10239-npr	CGCTTGGTGCAG GTCGGG	TCGCTTGGTGCAGGTCGGGAAATTACGATAGGTGTCAAGTGGA AGTGCA
MIRf10238-npr	TGCCTGGCTCCCT GTATGCCA	TGCCTGGCTCCCTGTATGCCACAATGTAGGCAAGGGAAGTCGG CAAAATGG
MIRf11025-akr	TGCATTTGCACCT GCACCT	TGCATTTGCACCTGCACCTTCTCATTACGATAGGTGTCAAGTGG AAGTGCA
MIRf10271-akr	TGGATTGAAGGG AGCTCT	TTTGGATTGAAGGGAGCTCTATGCCATGGTGGTCCTGCAGAGAT TATCGAACCTGGTATATCAGGGTTCCATATTGATCCA
MIRf10082-akr	TGCATTTGCACCT GCACCT	TGCATTTGCACCTGCACCTTCTCATTACGATAGGTGTCAAGTGG AAGTGCA
MIRf11010-npr	TTCCACAGCTTTC TTGAAC	TTCCACAGCTTTCTTGAACTTATTACGATAGGTGTCAAGTGGAA GTGCA
